# Collectanea

**Published:** 1844-03

**Authors:** 


### Collectanea.

*Medical Writers Good Practitioners.*—We copy the following pleasant article from the *Boston Journal.* The unjust and illiberal notion which the writer combats, is a very common one, and is encountered in the every-day walks of the physician. A still greater prejudice perhaps exists against the physician who cultivates some one or more of the natural sciences. The chemist, the botanist, or the mineralogist, seldom enjoys much patronage as a practitioner. To some extent there is a reason for it, as chemistry, botany, mineralogy and the kindred pursuits are so seductive, that he who becomes addicted to them is apt to *neglect* his professional avocations. But to the article:

An idea is prevalent in the world, and even fostered among medical men, perhaps from interested motives, that medical writers are not generally the best practitioners. The very reverse of this is more generally the fact. The nature of their researches obliges them to become familiar with the opinions and practice of the best practitioners, and to treasure them up with the most scrupulous attention. A store of useful and practical information is always at hand, which is ever ready to be applied to the cases under consideration and treatment. Writers, too, always provide themselves with large and respectable libraries for the purpose of investigating all the subjects on which they treat, as well as for the intrinsic value of the information they obtain from the perusal of them. Dr. Rush observes, "If a physician obtain skill by his own solitary experience, how much more will he acquire by availing himself of the experience of several hundred physicians, which he can only obtain by availing himself of the opportunity of perusing a large medical library." And if it be true, which can admit of but little doubt, that a physician cannot accurately remember the details of his practice more than three years, of how much importance is it that he should be in the habit of recording all important facts and cases which may occur to his notice and observation, and be continually treasuring up fresh stores of knowledge by unwearied attention to books. A physician's studies are never finished till the close of life. I was always very much pleased with the following anecdote of Dr. Rush. "As two young physicians were once conversing in his presence, one of them said—'When I *finished* my studies.' 'When *you* finished *your* studies!' said the doctor, abruptly; 'why you must be a happy man to have finished your studies so young! I do not expect to finish *mine* while I live.' "

Notwithstanding the above observations, we have too much reason to believe that a great proportion of practising physicians in America do not devote much of their time and attention to study after they commence the practice of their profession; and it is from this mental idleness, as Dr. Rush observes, that "it is no uncommon thing for an old physician (from his neglect of books) to be more ignorant than he was when he commenced the practice of his profession,"

Let it not be said that a physician has not time to record his experience or even to read. Some of the most extensive practitioners have been the most voluminous writers, and most industrious readers. In proof of this, we need only to mention the example of some of the fathers of our profession —such as Hippocrates, Galen, Celsus, Hoffman, Sydenham, Boerhaave, Van Swieten, Wistar, the Hunters, Monro, Cullen, and a host of others, almost all of whom were engaged in the most extensive and most lucrative practice, and were also authors of several most voluminous works. Dr. Good was one of our most elaborate writers. He has written several works, besides his great one on the practice of physic, yet the income of his practice was seven or eight thousand dollars a year. Sir Astley Cooper more than doubled the amount charged by Dr. Good in the same space of time; and yet, he was continually furnishing the world, through the medium of his writings, with the result of his knowledge and experience. Innumerable other examples in Europe might be mentioned of a similar nature. In fact, the best and most learned writers there, were altogether the best and most successful practitioners. Dr. Clark and Sir William Jones may be added to this list. They never for a moment neglected the duties of their profession. Indeed they excelled in the practice of that profession, and they were among the most eminent in Europe, in science and literature.

Our own country, too, is rich in examples to prove that our best practitioners are, and ever have been, our ablest writers. Among our deceased medical men we need only mention the immortal names of Rush, Barton. Redman, Wistar, Dorsey, Dewees, Physick, Parish, Ramsay, Miller, Hosack, Godman, Eberle, Warren, Gorham, and numerous others, whose writings alone would fill a decent library. Among the living we take great pleasure in enumerating the names of Jackson, Warren, Bigelow, Hale, Ware, Hayward, Shattuck, Smith, Holmes, Woodward, and many others in Massachusetts; of Parsons, Senter and others, in Rhode Island; of Tully, Ives, Sumner and others, in Connecticut; of Mott, Beck, McNaughton, Reese, Paine, Lee, Forrey, the Smiths, Delafield, *cum multis aliis,* in New York; of the venerable and learned Coxe, Samuel Jackson, Hays, Gerhard, the McClellans, Horner, Gibson, Bache, Dunglison, Wood, Bell, and innumerable others, in Philadelphia; not forgetting the names of N. R. Smith, Annan, Sewall, and others, at the South; and Gross, Drake, Cartwright, Mussey, Hildreth and Kirtland, beyond the Alleghanies. Let no one accuse me of partiality in this enumeration. I am sensible that I have omitted the names of very many who have been equally successful with their pens, and in their practice. My object was not to give a list of our celebrated writers, for that would fill a sheet, but to mention a few which, without much reflection, presented themselves to my mind in illustration of the truth of the opinion that our ablest and most elaborate medical writers are also our *very best practitioners.*

W. W.

January 8th, 1843. *Western Jour. Med. and Surg.*

---

*Influence of the Seasons.*—1. The amount of sickness in the central districts of London, during the year 1842, varied directly as the temperature, being a maximum in August, the hottest month of the year, and a minimum in January, the coldest month.

2. The diseases which determined the order of sickness, were febrile and catarrhal affections; the contagious, exanthemata, and the disorders of the digestive organs; to which may be added the mixed group, consisting of gout, scrofula, &c.

3. The diseases of the organs of respiration followed the inverse order of those already mentioned, and were inversely as the temperature, being most numerous in the colder, and fewest in the hotter months.

4. The temperature did not appear to exercise a marked influence on the other classes of disease: with the exception, perhaps, of those which form a measure of the activity of the sexual passion, which were in excess during the hottest months of the year—a fact which corresponds with, and corroborates, our experience of the influence of the seasons on crimes against the person, &c.

5. The hygrometric state of the air appeared to have little effect on disease; and, if it produced any effect, it was on the diseases of the organs of respiration, which were in excess during the months in which the quantity of moisture in the air was the greatest; but these were also the coldest months.

6. The mortality for the metropolis during the year 1842, was greatest in the first quarter, and least in the second, and was inversely as the sickness, except that the mortality of the third quarter exceeded that of the fourth.

7. The diseases which chiefly influenced the order of the quarters in respect of mortality, were those of the chest; to which may be added, as following the same order, the decay of nature in the aged. It is well known that the most common cause of death, in the aged, is an affection of the lungs, called "bronchitis senilis."

8. The order of the seasons in respect of sickness and mortality differs year by year, and does not admit of being reduced to any precise rule.

9. As a general rule, but one admitting of many exceptions, it may be stated, that the amount of sickness tends to vary directly, and the amount of mortality inversely as the temperature.

These results must be received with some reserve, as they are founded on a comparatively small number of facts; but they are, probably, not very far from the truth. At any rate they may prove suggestive of future inquiries, founded upon a broader basis. At present the materials for a comprehensive theory of the influence of the seasons and weather upon sickness and mortality are wanting, and are not likely to be supplied till the example set by one or two public hospitals and dispensaries shall have provoked imitation. In the meantime, the present attempt, if it accomplish no other purpose, may serve as an example of the mode by which such inquiries must be conducted.

In the course of this inquiry it is scarcely possible that some hypothesis should not have suggested itself as the most likely to prove true; and it may not be amiss to bring this attempt to a conclusion, by stating in a few words that which I have been led to form.

The causes of sickness are twofold, consisting of atmospheric changes which may be submitted to measurement, and of certain more subtle changes in the composition of the air, which, at present, can neither be analysed nor estimated. To the former class belong the temperature, moisture and pressure, of the air; to the latter those emanations from the earth, or from human beings themselves, which give rise to the majority of epidemic, endemic, and contagious diseases. As the number of cases of sickness produced by these latter causes, is generally considerable, the influence of the pressure, temperature, and hygrometric state of the air, will not be observed in those years in which these causes are in operation; but in the absence of epidemics, the temperature will be found to be the most influential cause of sickness. When the temperature of the summer is high, there will be such an amount of sickness in the summer months as to cause a large return of sickness for the entire year; so, on the other hand, a severe winter will swell the total sickness of the year, by producing a great excess of affections of the organs of respiration. A summer or winter of unusual length, beginning early and ending late, will also cause an increase of sickness on the entire year; but the nature of the sickness will be different as the temperature is higher or lower than usual. The order of the seasons in respect of sickness will also be mainly determined by the degree in which the temperature of these seasons exceeds, or falls short of, the average temperature.

The mortality, in like manner, in non-epidemic years, will be chiefly dependent upon the temperature, varying in the several seasons inversely as the temperature, except in those years in which the summer is unusually warm, when the mortality of the summer may even exceed that of the winter season. In other instances, the mortality of the summer months will rank next to that of the winter or autumn.—*Dr. Guy, in the Quarterly Journal of the Statistical Society.*

### METALLIC PASTES FOR FILLING TEETH.

*Royal Succedaneum, Enamel Cement, Bone Paste, Diamond Cement, Mineral Paste, Lithodeon.*—These are some of the names of a compound of mercury and other metals, by the use of which, for filling carious teeth, the public have been imposed upon again and again within the last thirty or forty years. With some slight variations, it has always been the same base article, under whatever name it has been presented.

I have always been unwilling to appear as an expositor of the abuses in dentistry which are at all times so much practised around us, except when they have become so excessive that I could keep silent no longer. And although I have witnessed the effects of this mercurial preparation for a long time, since its last introduction into our city and neighborhood, under one or another of the above imposing names, I have forebore to notice the article in this way till I should be fully satisfied, by repeated examinations, of its nature, and result of its application.

Testimony relative to these points has been so abundant, and has flowed in so fast, of late, that it would be a violation of duty and conscience not to speak out, and speak plainly concerning it.

Teeth filled with this *mercurial composition* are almost immediately changed in their complexion. Front teeth, in a few days after this *cement*

has been placed in them, become so blue or black as to be ruined in their appearance, while it is retained, even in cases where the anterior enamel is so perfect that a well-placed gold filling would not in the slightest degree change its natural healthy hue. Back teeth are often rendered so black, even into their fangs, that it is difficult if not impossible to restore them; and all this from the dark oxyd or salts of mercury which are formed from this metal in such a situation. Let one of these lumps of *cement* be removed after it has been placed in a carious tooth a few weeks, or in most cases in less than one week, and it will be found that its hidden surface, which was in imperfect contact with the tooth, will be as black as gunpowder—to say nothing of the offensive state of the tooth itself. But in addition to these effects, which are of the *least* consequence in the list, there follow pain, swelling, gumboils, ulceration, inflammation extending to adjacent teeth, swelling of the glands about the tongue, throat and neck, neuralgia about the jaws, face and temples; and where several large fillings are placed at about the same time in very hollow teeth, even *salivation* is produced in those who are highly susceptible to the influence of *mercury*. All these are effects which I have either witnessed repeatedly, or of which I have obtained accounts from the most respectable dentists in our country. I am even now called from writing, to examine a case—the effects of a large filling of "*lithodeon*," in which the under surface of the tongue is constantly irritated, and has been several times ulcered by coming in contact with the mercury. And I have a collection of specimens—teeth that that have been extracted, charged with "lithodeon"—which will fully illustrate the above statement; for I have found it requisite to extract more adult teeth in the course of the last two or three years, on account of the mischievous effects of *mercurial paste*, than for any one other cause, sufficient time having elapsed, since its last introduction here, to show, not the immediate bad consequences, but very many of the remote.

The testimony of Dr. E. Parmly—a gentleman of high professional reputation in the city of New York—should have much weight in relation to this matter. He has in several instances expressed his opinion publicly concerning it. His language, as quoted in Maury's Dental Surgery, p. 152, is—

"For this operation" (the filling of teeth) "gold is the only substance known that can be permanently relied upon; although there are cases in which tin, and even lead, may be of temporary service when employed with skill and judgment. I regard cements, fusible metals, amalgams, succedaneum, and all other substitutes for the above-named metals, as impositions on the public, never having seen a single operation in which these substances were employed, which would not have been more permanent, if even lead, the poorest of these metals, had been used; because it is less subject to decomposition and oxydation, to say nothing of the poisonous qualities of the mercury which most of the others contains. I have never known a perfect master of the art of stopping teeth either to employ or recommend the substances which I here condemn; and I believed the use of them is almost wholly confined to those persons who are unacquainted with this nice and difficult art."

This mercurial compound is still in use in our city and the country about it, I will not say by dentists, but by a host of impostors, "operators on teeth," whose advertisements fill a part of almost every newspaper; some of whom perhaps are even ignorant of its deleterious effects, but many of whom know well its qualities, and too well to trust it in their own teeth. It is an article which can be applied by any who can stop a hollow tooth with wax or putty, and if it could be retained no longer than these, its evils would be very greatly diminished.

I am fully aware that these *cements* or *amalgams* have been used in some cases where they *seem* to be of service; but here, still, is deception; for in all such that have come under my observation, (and these are very numerous,) it can be demonstrated, by an examination of them, that great mischief is going on beneath such fillings, and that a different and better treatment might have been adopted. J. F. Flagg.

*Boston, Dec. 5th,* 1843. *Boston Med. and Surg. Jour.*

*The Blue Lick Springs of Kentucky.*—Through the entire mountain ridges of Virginia, the medicinal waters are gushing out at various points in copious streams; but some few locations have more reputation than others, which they will doubtless maintain for generations to come. In the state of Kentucky, there are fountains equally distinguished for their properties in subduing a variety of diseases, yet their geological relations are quite different from those on the lofty elevations of the Alleganies. They bubble up in peculiar depressions on the line of shallow ravines, technically called, in the vernacular of the country, *licks*. There are the *Blue Licks, Big-bone Licks,* &c. &c., well known to the traveller, the geographer, and those in pursuit of health.

From a remote antiquity, the wild animals of the forest traversed the whole region of country to gratify a strong and perhaps innate propensity for salt, which was held in solution in the water that was forced out of the ground at the licks. Nearly the whole if not all the licks are saline springs. There, countless herds of buffaloes, the deer, and perhaps all the smaller animals, met at one common rallying place, to lick their mother earth, saturated with marine salt. There, they probably contended for the right of possession, warring upon each other from age to age, in the solitary gloom of a mighty forest, undisturbed by the footsteps of man, and there accumulations of skeletons were left to bleach and decay—the memorials of brute warfare. But long before—even at an epoch so distant that geology itself furnishes no data for a probable conjecture in respect to the period—the mastodon, colossal in size, and powerful in strength, sought the same licks, and gratified the same indomitable appetite for salt that characterizes the races that have been ushered into being since its gigantic bones have been fossilizing under the feet of a new order of animal organization.

There is neither exaggeration nor extravagance in these observations, however much the relations of naturalists may excite the marvellousness of the unlearned in this rich but poorly cultivated domain of natural science. Within the last six weeks, some extensive improvements have been in progress at the Lower Blue Lick Spring, requiring an enlargement of the well, in order to line it with stone. In digging only about three feet from the water, laterally, five feet below the surface, the tusk of a monster animal was uncovered, six feet two inches in length, by twenty-two inches in circumference, some portion of which is still in a tolerable degree of preservation. There were also several enormous grinders, that might have remained there, for aught that is known to the contrary, till the elements shall melt with fervent heat, unchanged in form, and unaltered by the corroding influence of time. These extraordinary trophies, so recently discovered that their advent has not before been chronicled, now lie on the office floor of the hotel, for the inspection of visitors. Such bones are unquestionably lying but a few feet below the soil, over the whole basin of the lick.

On the Lexington turnpike, twenty-four miles from Maysville, Ky., is the *Lower Blue Lick Spring*—a fashionable resort for persons of leisure, during the sultry months of summer, and celebrated for its many medicinal properties, and consequently a great focus to which invalids concentrate from all parts of the United States. After passing through several hands, it has finally come into the possession of two or three brothers, who are conducting the establishment on a scale of elegance very satisfactory to the peripatetic public. The Spring is leased for ten years at an annual rent of $1500, with a right to a deed by the payment of $25,000 at the expiration of the contract. Large quantities of the water are sent off in barrels, at $1 a barrel, but $2 is asked if the casks are furnished by the proprietors. It is said that the sales have amounted to $4000 in the last two months. Such is the increasing demand, that New Orleans, Mobile, Natchez and Cuba have become extensive markets for it. Agencies are being established so actively, that it is fully believed the annual sales may reach as high as $12,000 within a few years.

When the water was used expressly for manufacturing salt, it required 800 gallons to yield one bushel—being in the proportion of 1 to 80. According to Dr. Yandell's analysis, it contains—sulphuretted hydrogen, 2; carbonic acid, 3; muriate of soda, 4; muriate of magnesia, 5; muriate of lime, 6; sulphate of lime, 7; sulphate of soda, 8; sulphate of magnesia, 9; carbonate of lime, and probably, says that able chemist, a trace of carbonate of magnesia. In its action upon the system it is purgative, diaphoretic, diuretic, and alterative, and stands highest in rank in the catalogue of salino-sulphureous waters. On the report of the same gentleman, it is identical with the Harrowgate waters of England. With respect to its effects on the system, according to the representation of its friends, all maladies yield to its sovereign influence. They melt away under its potent agency, like the odor of plants on the wings of a zephyr. It blows hot or it blows cold: in a word, it gently brings down the overgrown rotundity of a gourmand, or fattens the frail tenement of his antipode in dimensions. It is the misfortune of all mineral waters, in this country, either to be overrated by the incompetent, or underrated by physicians. When medicine has been judiciously prescribed for a considerable season, without manifest advantage, in certain obscure visceral, or perhaps cutaneous, affections, the patient not unfrequently posts off to a spring. There he drinks, till a restoration has been accomplished, not by the course he is pursuing, but mainly by the action of the very medicinal treatment that is overlooked—the exercise of a strong faith in the all-healing influences of the fountain. Better analyses are wanted. A more careful series of observations by medical men than have yet appeared, and a proper reliance on common sense and the wisdom of judicious practitioners, are also needed by those who hope for the most in visiting thermal or the ordinary mineral waters of any place.

Nothing short of miracles are ordinarily performed at the onset, in the estimation of new comers—an error that is soon corrected when the old malady peeps out again, after it was exorcised by the potency of a celebrated water. Finally, we have come to the conclusion that our real knowledge in regard to the true medicinal value of these waters, is very small indeed. There is abundant room for inquiry, and an imperfectly explored field is before the world, however much it may be bruited that nothing further remains to be investigated in relation to them. So many persons are interested in them as properties, that many obstacles are in the way of testing points of considerable moment; but after all, were the springs not the centres of fashionable life, where strangers, the elite from every community, far and near, concentrate to eat, drink, dance and be merry, they would at once be bereft of their reputation, and ultimately sink into utter oblivion, save in the estimation of those who might seek them from a higher motive, to be cured of a real and not imaginary disease.

Hoping hereafter to notice the characteristics of the White Sulphur, the Red, Blue and Sweet Springs of Virginia—the Warm and Hot being already partially disposed of—we shall then give attention to the Harrodsburg and Big Bone in Kentucky.—*Boston Med. & Surg. Jour.*

*Compliment to the Memory of Sir Charles Bell.*—Sir Robert Peel has addressed the following letter to Lady Bell:—

*Whitehall, September* 4.

"Madam,—I have had great pleasure in recommending to Her Majesty, that in consideration of the high attainments of your lamented husband, and the services rendered by him to the cause of science, a pension of one hundred pounds per annum for your life, shall be granted to you, from that very limited fund which Parliament has placed at the disposal of the Crown for the reward and encouragement of scientific labors.

This pension, small in amount as it necessarily is, will perhaps be acceptable to you, as a public acknowledgment on the part of the Crown, of the distinguished merit of Sir Charles Bell.

I have the honor to be, Madam, your faithful and obedient servant,

ROBERT PEEL."

